# Medical History from the Earliest Times

**Published:** 1893-04-22

**Authors:** E. T. Withington


### Medical History from the Earliest Times.

By E. T. Withington, M.A., M.B. Oxon.

### LI.—THE REFORM OF SURGERY.

The progress of anatomy not only paved the way for Harvey, but brought with it a new surgery, founded no longer on Galen and Albucasis, but on nature and experience. Many of the distinguished anatomists already mentioned—Berengar, Vidius, Fallopius—were also able surgeons, but it will be best to confine ourselves to four typical representatives of the art—Ambrose Paré (1517-90), Peter Franco (1505-70 ?), Gaspar Tagliacozzi (1545-99), and Fabricius Hildanus (1560-1634).

The first of these, justly called the Father of French surgery, rose from the lowest rank of his profession to be Councillor of State and surgeon to four Kings of France, and enriched his art both by the restoration of old and the invention of new modes of treatment. Something of his character, and one of his most important innovations, may be learnt from the following shortened story of his first campaign. In 1536, his twentieth year, Paré went as surgeon of infantry with the expedition to Turin. In the first engagement a Captain Le Rat was wounded. "I dressed him, God cured him," says Paré, a phrase which he repeats elsewhere, and which is now engraved upon his statue. On entering Susa he found three badly wounded men in a barn. "Can these men live?" asked an old soldier who followed him, and when Paré said "No," he went up to them and cut all their throats sweetly and without wrath (doucement et sans cholére). The young surgeon was horrified, but soon had other things to think of, for in the next fight there were so many wounded that the boiling oil with which gunshot injuries were then treated, in the belief that they were poisoned, was exhausted, and Paré was obliged to resort to a simple dressing. He describes his anxiety during the night as to the fate of those thus treated, and his amazement at the favourable result, which determined him never to use the old treatment again. Among Paré's other services are the reintroduction of ligature in amputation, and of podalic version in obstetrics; the invention of improved forms of surgical instruments; the distinction between fractures of the neck and shaft of the thigh bone; and his notice of the fact that sound teeth accidentally knocked or pulled out may be successfully replaced in their sockets. Paré, like Paracelsus, wrote in his native tongue, but though a reformer, he was no revolutionist, and one of his aphorisms, "A tried remedy is better than one newly invented," may still be remembered with advantage.

The earliest known account of what is, perhaps, the most important of surgical operations (that for the relief of strangulated hernia) was published by Peter Franco in A.D. 1556. It was repeated by Paré in 1575, and by Rousset in 1581, neither of whom condescends to mention Franco, for he belonged to a class who were considered unworthy of the name of surgeons, the travelling lithotomists. Peter bitterly laments the low estate of his fraternity: "The physicians and surgeons, even the apothecaries, can defend themselves when they are unfortunate, but if we have a mishap we must often run for our lives." Some of his colleagues, he admits, deserve punishment as much as highwaymen, "for they fear neither God nor man, but plunder and torture their patients on pretence of benefiting them." His skill, however, soon raised him to a higher rank, and he became one of the salaried surgeons of the Republic of Bern. Franco is most widely known as the first performer of suprapubic lithotomy, and his works, though written in an illiterate style, contain the clearest descriptions of surgical procedures to be met with since classical times. His description of the symptoms of "stone" is especially good, but we have only space to give the classification. "In phlegmatic persons stones are usually white, smooth, and grow rapidly (phosphates). In the melancholic they are black, rough, hard, and grow slowly (oxalates). In the choleric they are reddish and of rapid growth, but sometimes soft (urates)."

The fame of Tagliacozzi, Professor of Surgery at Bologna, rests upon a single operation, that of rhinoplasm, or the manufacture of new noses. This feat, originally achieved by the Hindus, had been accomplished in another manner in the preceding century by certain Sicilian and Calabrian surgeons, who practised it as a family secret. Perhaps the earliest existing notice of the operation is the following letter which Calentino, a Neapolitan poet, wrote in 1442 to his friend Orphian, who had lost his nose: "If you want a new nose pay me a visit. Branca, a Sicilian surgeon, has found a way to restore lost noses. He either takes flesh from the patient's arm, or engrafts on him a slave's nose. The thing is truly marvellous! As soon as I saw it I made haste to send you the news, for to whom could it be more important? Rely upon it, if you come hither, you can go away with as many noses as you like." To Tagliacozzi, however, belongs the credit of first fully describing the operation, of investigating the nature of the process, and of carrying it out with the most brilliant success. His fame as a restorer of noses spread throughout Europe, and Bologna was filled with his patients. But this success was not without alloy; he was bitterly attacked by the same class of theologians, who afterwards opposed as impious the introduction of inoculation for small-pox, and the use of chloroform in obstetrics. They now accused Tagliacozzi of impiously presuming on the function of the Creator, and attributed the success of his operation to the assistance of the devil. And their hostility pursued him even to the grave. For many nights after his burial the nuns of a neighbouring convent heard a terrible voice, which cried, "Tagliacozzi is damned!" and the clergy of Bologna thereupon ordered the great surgeon's body to be cast out of the church and buried in unconsecrated earth. His colleagues, however, raised him a statue in the anatomy school, where he stands immortalised, a nose in his hand.

William Fabry, of Hilden, near Dusseldorf, whose name is recorded in medical history as Fabricius Hildanus, was worthy to have been the Paré, or even the John Hunter of Germany; but he was unfortunate in the time of his birth, for the good seed he sowed fell on fields already planted with the tares of Paracelsic mysticism, and destined to be ploughed by the cannon of the thirty years' war. Though without a University education, he had gained an intimate knowledge both of Greek and Latin, and it was the object of his life to raise surgery to the dignity of a science, and the surgeon to the rank of the physician. To this end he continually urges the student to keep abreast of the increasing knowledge of the day, especially in anatomy, "the key, compass, and foundation of medicine," a science which should be studied not only by physicians and surgeons, but by the clergy and magistrates; in short, by all who have to guide or govern mankind. The surgeon should also study botany and chemistry, "for metallic remedies are of great value in medicine, as we daily experience, though when given by quacks and 'idiotæ,' they are like a sharp sword in the hands of a fool, or a lighted torch held by a child"; probably a reference to the Paracelsists, who were his special abomination. At Bern, where Fabricius was appointed city surgeon, he kept a private hospital, and gave clinical instruction to students and practitioners, while, like Hunter, he founded a museum containing not only anatomical and pathological preparations made by himself, but archæological specimens, the thigh bone of a mammoth and other curiosities. He was especially skilful in inventing new instruments, such as aural specula, splints, and forceps for removing foreign bodies, and he even made an artificial eye, which he presented to his pupils, to encourage them in their anatomical studies. But the reader should refer elsewhere for an account of the life and works of one who is perhaps the most attractive of the four great surgeons here briefly noticed, and we must conclude with an extract from his chief work, the "Six Hundred Surgical Cures and Observations." On April 25th, 1624, he writes to a Dr. Hagenbach, regretting that an attack of gout prevented him from going to the latter's wedding, and relates the following case as an example of the advantages of being married. "A countryman, Benedict Barquin, bought some iron, and was striking two pieces together to prove its quality, when a splinter flew into his eye and stuck in the cornea, causing him great pain. The local surgeons tried everything for many days to no purpose, and the pain and inflammation so increased that he came to me at Bern on March 5th. I used all means I could think of for some days, but the splinter was so small that it could not be removed by instruments. When, behold! my wife hit on the very thing. I kept the eye open with both hands, while she held a magnet as close as possible to it, and after several trials (for he could not stand the necessary light long) we saw the iron leap from the eye to the stone." This ingenious lady was a French Swiss from Geneva, named Marie Colinet, who, in her husband's absence, could not only treat diseases of her own sex, but even cases of fractured ribs and legs.

